# Awareness of and willingness to use pre-exposure prophylaxis (PrEP) among people who inject drugs and men who have sex with men in India: Results from a multi-city cross-sectional survey

**DOI:** 10.1371/journal.pone.0247352

**Published:** 2021-02-25

**Authors:** Ashwin Belludi, Allison M. McFall, Sunil Suhas Solomon, David D. Celentano, Shruti H. Mehta, A. K. Srikrishnan, M. Suresh Kumar, Suniti Solomon, Gregory M. Lucas

**Affiliations:** 1 Department of Epidemiology, Johns Hopkins Bloomberg School of Public Health, Baltimore, Maryland, United States of America; 2 Department of Medicine, Johns Hopkins University School of Medicine, Baltimore, Maryland, United States of America; 3 Y.R. Gaitonde Center for AIDS Research and Education, Chennai, Tamil Nadu, India; Washington University in Saint Louis, UNITED STATES

## Abstract

**Introduction:**

Pre-exposure prophylaxis (PrEP) is effective in reducing HIV transmission among key populations. In India, where PrEP is not currently part of the national HIV program, little is known about PrEP awareness, willingness to use PrEP, and barriers to uptake among people who inject drugs (PWID) and men who have sex with men (MSM).

**Methods:**

We used respondent-driven sampling to accrue PWID and MSM in 22 sites from August 2016 to May 2017. Participants were asked about awareness of PrEP, willingness to use PrEP (following a brief description) and reasons why they might not be willing to use PrEP. Participants were also queried on preferences for PrEP delivery modality (oral vs. injectable). Multi-level logistic regression models were used to determine participant correlates of willingness to use PrEP. Estimates were weighted for the sampling method.

**Results:**

A total of 10,538 PWID and 8,621 MSM who self-reported being HIV-negative were included in the analysis. Only 6.1% (95% confidence interval [CI]: 5.9, 6.3) of PWID and 8.0% of MSM (95% CI: 7.7, 8.4) were aware of PrEP. However, willingness to use PrEP was substantially higher in both groups: 52.4% of PWID and 67.6% of MSM. Participants commonly cited a perceived low risk for acquiring HIV infection, being perceived by others as being HIV-positive, and side effects as reasons why they would be unwilling to use PrEP. Among PWID, sharing needles and hazardous alcohol use were associated with increased willingness to use PrEP. Among MSM, having a main male partner and injection drug use were associated with increased willingness to use PrEP. Preference for daily oral or monthly injectable PrEP was similar among MSM (39.6%% vs. 41.7%,), while PWID were more likely to prefer oral to injectable administration routes (56.3% vs. 31.1%).

**Conclusions:**

As India plans to roll-out of PrEP in the public sector, our multi-city survey of PWID and MSM highlights the need for key population-focused education campaigns about PrEP and self-assessment of risk.

## Introduction

Since the first identification of HIV-infected individuals in 1986, India has made substantial progress in controlling the HIV epidemic, particularly with respect to heterosexual transmission [1]. Recent data released from the National AIDS Control Organization (NACO) for 2017 estimated HIV prevalence among adults (15-49 years) to be 0.22% and the number of new infections to be approximately 87,000 per year. This represents an 85% decrease in the HIV incidence in the general population compared with 1995 [1, 2] – however, the decline seems to have plateaued over the past few years highlighting the need for new strategies to end the HIV epidemic in India.

Further, the declines have not been uniform across all risk groups. HIV prevalence and incidence remain high in key populations, including people who inject drugs (PWID) and men who have sex with men (MSM). The estimated average HIV prevalence among PWID and MSM in India are 6.3% and 2.7%, respectively [3–6]. We recently reported average HIV incidence rates, across the 22 sites in this analysis, of 5.2 and 1.4 per 100 person-years among PWID and MSM, respectively [7]. Use of banned narcotic drugs is a criminal offence under Indian legal system as is injection of these drugs. Injecting drugs and same-sex behavior are stigmatized in India. Additionally, sex between men was illegal under the Indian Penal Code until the law was overturned by the Supreme Court in 2018 [8–10]. There is increasing recognition that traditional strategies of prevention such as promotion of safe sexual and injection practices, syringe service programs, and free condom distribution through government programs must be supplemented by new strategies [11].

The landscape of HIV prevention has changed with the advent of pre-exposure prophylaxis (PrEP) [12]. PrEP is a biomedical tool that is part of a multi-faceted, comprehensive approach to prevent HIV transmission. A recently published analysis found that PrEP was likely to be cost-effective among PWID and MSM in India, across a broad range of assumptions [13]. India is committed to the goal of “End AIDS as a public health threat by 2030” and has included PrEP as part of the National Strategic Plan for HIV/AIDS and STI 2017 – 2024 to pave the way for a ”AIDS Free India” [14]. Additionally, India has a licensed co-formulated product approved for marketing by the Drug Controller of India in May 2016 [15]. However, PrEP is not yet provided for free as part of the national program, though guidelines are currently under development. Studies done in limited geographies of India indicate that there is low awareness and higher willingness to use PrEP in India, but these were done among MSM and transgender individuals (TG) and the sample sizes were small [16, 17]. There is still paucity of information about PrEP awareness and willingness to use PrEP from other geographies of India and among PWID. Thus, we assessed awareness of and willingness to use PrEP, and correlates of willingness to use PrEP, in PWID and MSM from 22 sites across India. Our findings will provide context and assist stakeholders as they plan for the implementation of public sector-supported PrEP in India.

## Materials and methods

### Background and setting

**Fig 1:**
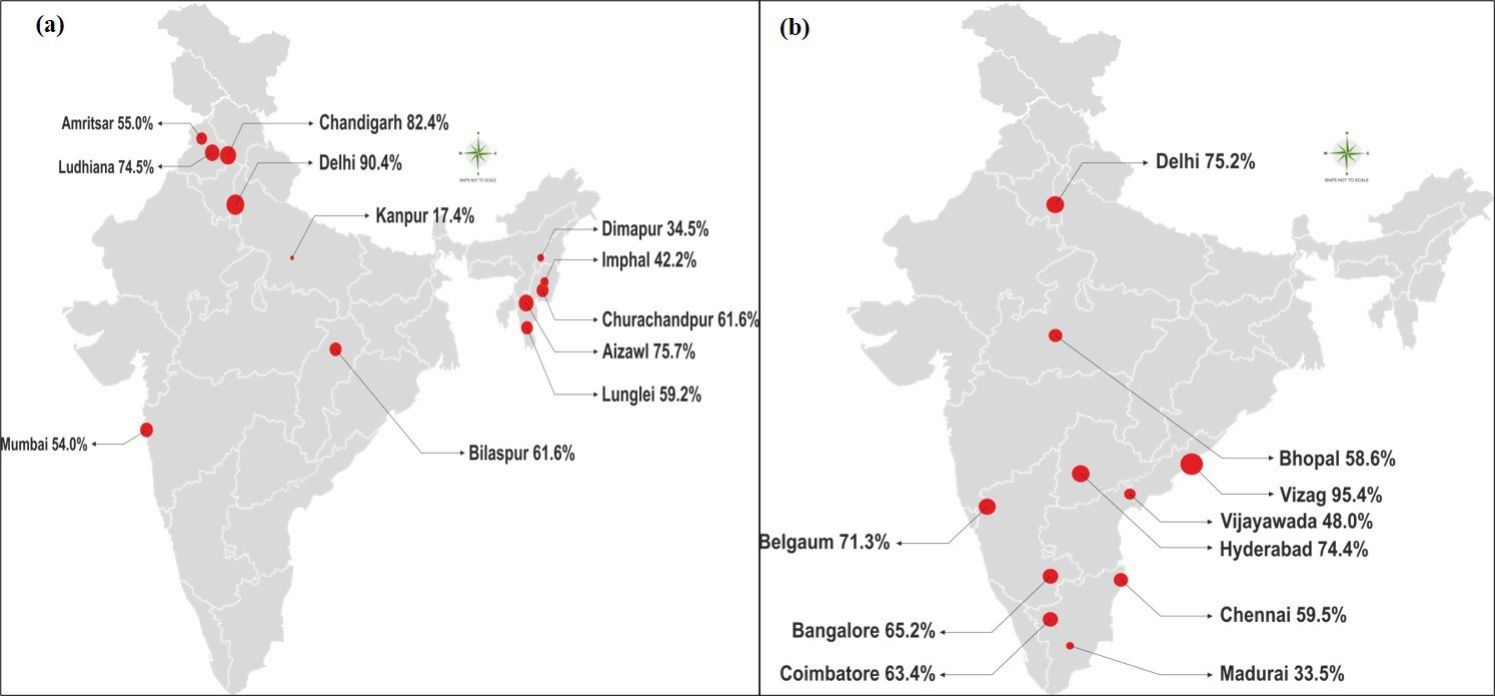
Percentages of PWID (a) and MSM (b) who endorsed willingness to use oral pre-exposure prophylaxis (PrEP), by city. The circle sizes at each city are proportionate to the percent willing to use pre-exposure prophylaxis. Estimates include sampling weights for respondent-driven sampling.

The present study is a secondary analysis of data collected from a cross-sectional survey of PWID and MSM across 22 sites in India. The survey was conducted as part of a cluster-randomized trial (ClinicalTrials.gov: NCT01686750) that evaluated the effectiveness of integrated care centers to increase HIV testing, access to risk reduction services, and linkage to care among HIV-positive PWID and MSM [7]. The key populations and cities were selected, in consultation with NACO, based on preliminary evidence of high transmission risk or poor engagement with the HIV care continuum [3]. The 12 PWID sites and 10 MSM sites are shown in **Fig 1**(Fig 1A for PWID and 1b for MSM). Delhi was the only city where both PWID and MSM were surveyed, and there were separate study sites for the two populations. During the time frame of the survey (2016-17), PrEP was available in the private sector in India, but was not available through government-sponsored programs. The intervention being assessed in the parent trial did not provide PrEP itself or information about PrEP.

### Study design

We used respondent driven sampling (RDS) to recruit PWID and MSM in the specified sites. RDS is a peer chain-referral strategy that is effective in accessing hidden populations and, under certain assumptions, yields unbiased population estimates when RDS weights are applied [18]. We used 2 or 3 “seed” participants (influential and well-connected members of the population) to initiate recruitment in each city [3]. Seeds and subsequent participants were provided two bar-coded referral coupons to recruit their network members. This process was continued until the target sample size (n~1000) was reached in each city, with the exception of Mumbai (n = 724), which had to close participant recruitment early due to slow enrollment. A biometric system was used to ensure individuals did not participate in the survey more than once. Eligible participants were reimbursed INR 250 (USD 3.47) for completing the survey and INR 50 (USD 0.69) for each eligible participant they recruited to the study via the coupon system (up to 2). Individuals were eligible to participate if they were at least 18 years of age, had a valid RDS referral coupon (except for seeds), were able to comprehend English, Hindi or any of the seven additional regional / local languages, and reported the relevant risk behavior (a biological male reporting oral or anal sex with another man in the prior year [MSM] or a man, woman, or transgender person reporting injection drug use in the prior 2 years [PWID]). We did not ask participants whether they had used PrEP as we expected PrEP use to be rare in this population in the absence of a publicly funded program.

### Study procedures

Study procedures included an interviewer-administered questionnaire, a blood draw, rapid HIV testing (after the questionnaire), and pre- and post-test counseling. Detailed information on study procedures have been published elsewhere [3]. The questionnaire included questions about knowledge and attitudes towards PrEP (Supplemental material). First, participants were asked whether they were aware of (i.e., had ever heard of) PrEP as a way to prevent HIV. After a brief description of oral PrEP to participants who were unaware of PrEP, participants were asked whether they would be willing to take PrEP every day to reduce their risk of acquiring HIV (responses: no chance, very little chance, undecided, some chance, and very good chance). Participants were characterized as willing to use PrEP if they answered some or very good chance. Participants who answered undecided, very little chance, or no chance were asked to select reasons why they would not be willing to use PrEP. Participants were asked similar questions about a hypothetical injectable PrEP formulation, which would require injections every one to two months. Subsequently, they were asked whether they would prefer PrEP taken as a pill every day or as an injection every one to two months, excluding individuals who said they were not interested in either form of PrEP. To explore the roles of reported HIV risk behaviors and self-perceived HIV risk in willingness to use PrEP, we categorized respondents into three groups: i) willing to use PrEP, ii) unwilling to use PrEP and endorsed low perceived HIV risk as a reason, and iii) unwilling to use PrEP and did not endorse low perceived HIV risk as a reason. We compared demographic factors and HIV risk behaviors in the three groups.

### Statistical analysis

Seeds and participants who reported an HIV-positive status in the questionnaire were excluded from the analysis [19]. The RDS-II estimator, which weights individuals based on their network size, was used to calculate city-level population characteristics (e.g., percent aware of PrEP). Participants were asked to estimate their network size (i.e., number of PWID/MSM they had seen in the prior 30 days) in the questionnaire. For population estimates that pooled all PWID or MSM cities, a composite weight was used which included the individual RDS-II weight and a weight for the underlying population size (PWID or MSM) in each city. We used Spearman correlation coefficients (ρ) and associated p-values to assess city-level correlations between potential explanatory factors (i.e., awareness of PrEP and HIV prevalence) and willingness to use PrEP.

We used univariable multi-level logistic regression models with random intercepts for city to assess individual-level associations between participant characteristics and willingness to use PrEP, resulting in odds ratios. We then ran multivariate models in which we included demographic characteristics (age, sex (PWID), sexual identity (MSM), education, marital status, income) in addition to variables that were significantly (P <0.05) associated with willingness to use PrEP in univariable weighted analysis. Models were conducted separately for PWID and MSM. Those who responded either ‘don’t know’ or refused to answer the willingness question were excluded. Correlates assessed in both populations included age, marital status, education, household income, hazardous alcohol use (Alcohol Use Disorders Identification Test [AUDIT] score of 8 or above [20]), number of sexual partners, sex work, unprotected sex in prior 6 months, recent HIV-positive injection or sex partner, HIV test in prior 12 months, and a composite stigma score which measured key population stigma (PWID or MSM) rather than HIV or PrEP-related stigma [21]. The Cronbach’s alpha statistic was 0.90 for PWID and 0.96 for MSM. Correlates assessed only among PWID included sex, needle/syringe sharing, use of syringe service program, use of opioid agonist treatment, incarceration in prior 6 months, and ever MSM behavior (men only). Correlates assessed only among MSM included sexual identity, penetrative/receptive anal intercourse, having a main male partner, symptoms of sexually transmitted infection, drug injection, syphilis infection, and herpes simplex virus type 2 (HSV-2) infection (the latter two factors were assessed by serum testing).

This study was conducted and reported in accordance with the Strengthening the Reporting of Observational Studies in Epidemiology – Respondent Driven Sampling (STROBE-RDS) guidelines [22]. Main analyses were weighted; unweighted analyses are presented in Supplemental Material. We used Stata software (version 15.0; StataCorp, College Station, Texas, USA) for analyses. P-values <0.05 were considered statistically significant.

### Ethical oversight

This study was approved by the institutional review boards of YRGCARE in Chennai, India, Johns Hopkins Medicine and Johns Hopkins Bloomberg School of Public Health in Baltimore, Maryland, USA. Participants provided oral consent.

## Results

### General characteristics of PWID and MSM

A total of 11,745 PWID and 10,025 MSM were recruited via RDS (August 2016-May 2017). Of these, 10,538 PWID and 8,621 MSM did not self-report an HIV-positive status (i.e., they reported their HIV status as either negative or unknown) and were included in the analysis. Across the 22 study sites, the median (range) number of RDS recruitment waves was 14 [9–27], and median time to complete enrollment was 154 days (95-269). Equilibrium for the main factor of interest (i.e., PrEP awareness) was reached in all sites with the exception of one, Coimbatore (MSM), in which there were 9 waves of recruitment and awareness was still increasing (38.3% in wave 8 to 41.4% in wave 9) [7]. All participants responded to the question about PrEP awareness, while 9,836 (93%) PWID and 8,514 (99%) MSM responded to the question about willingness to use PrEP. Pooled characteristics for PWID and MSM have been summarized in Table 1 for weighted estimates and S1 Table for unweighted estimates. Site-level data are presented as the value at the median site, followed by the site range, an approach we have followed previously, as the survey was conducted in multiple sites with high city-to-city variability [7, 23, 24]. The site median for age ranged from 25 to 35 years among PWID and from 21 to 31 years among MSM. Recent sharing of needles/syringes ranged from 2.7% to 59.1% among PWID sites and recent unprotected anal sex ranged from 17.9% to 71.6% among MSM sites. Although, participants who reported HIV-positive status in the survey were excluded, HIV prevalence (based on point-of-care testing after the survey) ranged from 2.5% to 33.1% and 0.7% to 10.9%, at PWID and MSM sites, respectively.

**Table 1:** Characteristics of people who inject drugs and men who have sex with men who participated in a respondent-driven sampling survey across 22 sites in India between August 2016 and May 2017.

****Characterisitic****^1^	****PWID (12 sites, 10538 individuals)****	****MSM (10 sites, 8621 individuals)****
Age, years, median (site range)	28 (25-35)	28 (21-31)
Female	1.2 (0.5-20.7)	--
MSM identity		
*Panthi*^*2*^		42.8 (10.1-86.8)
*Kothi*^*2*^		12.9 (4.6-49.4)
*Double-decker*	--	25.3 (4.9-52.1)
MSM/gay		0.8 (0-6.2)
Bisexual		2.9 (0-32.6)
Marital status		
Never married	48.3 (31.5-57.6)	55.9 (11.9-73.0)
Married/living with partner/long-term partner	42.3 (25.6-60.5)	41.1 (25.3-87.4)
Widowed/divorced/separated	9.3 (1.5-24.9)	2.1 (0.7-9.5)
Completed secondary school	69.3 (36.3-97.9)	85.7 (71.7-91.7)
Household monthly income (INR), median (site range)	15,000 (9,000-30,000)	15,000 (10,000-20,000)
Incarceration in prior 6 months	5.8 (2.6-24.2)	2.2 (0.01-8.6)
Hazardous alcohol use^3^	39.3 (8.6-54.6)	34.5 (8.5-43.8)
Injected drugs in prior 6 months	86.3 (30.3-99.1)	0.3 (0-2.8)
Shared needle/syringe in prior 6 months	33.2 (2.7-59.1)	0
Syringe service program used in prior 6 months	20.7 (2.7-50.8)	--
Opioid agonist treatment used in prior 6 months	25.6 (0.9-52.6)	--
Number of sexual partners^4^ in prior 6 months, median (site range)	1 (0-1)	1 (0-5)
Unprotected sex^5^ in prior 6 months	46.1 (24.4-73.3)	59.8 (17.9-71.6)
Sex work in prior 6 months	1.5 (0.1-12.6)	15.4 (6.6-38.5)
HIV test in prior 12 months	33.5 (3.4-52.6)	27.7 (9.1-41.4)
Drug use/MSM behavior-related stigma^6^, median (site range)	7.3 (1.9-11.4)	3.3 (0-8.1)
Active syphilis infection^7^	--	6.4 (1.5-10.9)
HSV-2 seropositive^7^	--	25.1 (18.1-36.8)
HIV-positive^7^	11.1 (2.5-33.1)	4.0 (0.7-10.9)

PWID, people who inject drugs; MSM, men who have sex with men; HSV-2, herpes simplex virus type 2. INR, Indian rupees (exchange rate INR 72: USD 1).

^1^Data shown as percent at the median site (site range), unless otherwise noted. Estimates are RDS-II weighted.

^2^*Panthi* and *kothi* refer to masculine and feminine sexual identities, respectively. Men who self-identify as panthi tend to have masculine mannerisms/ appearance and may mainly or only engage in penetrative intercourse with men. Men who self-identify as kothi often show more feminine mannerisms/ appearance and mainly or only engage in receptive anal intercourse with other men. Men who self-identify as Double-decker may have feminine or masculine mannerisms/appearance and engage in both receptive and penetrative intercourse with men. Men who self-identify as gay, bisexual or MSM have fluid identities / behaviors that change over time.

^3^Hazardous alcohol use defined as an Alcohol Use Disorders Identification Test (AUDIT) score ≥8.

^4^Male and female sexual partners for PWID, male sexual partners for MSM.

^5^Vaginal or anal sex for PWID, anal sex for MSM.

^6^Composite stigma score including sub-scales: enacted, vicarious, felt normative, and internalized stigma; range: 0-20, with higher scores indicating more stigma. The scale had accepted reliability with Cronbach’s α = 0.90 for PWID and 0.96 for MSM).

^7^According to serum testing done at the survey visit.

### Awareness and willingness to use PrEP among PWID

In analyses of data pooled across cities, awareness of PrEP was low among PWID (6.1%, 95% confidence interval [CI]: 5.9, 6.3) with more than 10% awareness being observed in three PWID cities (Churachandpur [30%], Ludhiana [19%] and Lunglei [11%]). Despite low awareness, a substantial percentage of PWID expressed willingness to use oral PrEP. In analyses of data pooled across sites, 52.4% [95% CI: 51.8, 53.0] of PWID were willing to use oral PrEP with substantial heterogeneity in willingness to use oral PrEP observed across cities. (Fig 1A; range: 17.4%, 90.4%). Willingness to use oral PrEP at the city-level was not significantly associated with the level of PrEP awareness in the target population among PWID (ρ = 0.34, P = 0.28). Unweighted estimates of awareness and willingness to use PrEP are shown in S2 Table. There was no evidence that site allocation to the intervention or the control condition was associated with PrEP awareness or willingness to use PrEP (data not shown).

Willingness to use an injectable formulation of PrEP tracked similarly with the data for oral PrEP, with 49.0% (95% CI: 48.5, 49.5) of PWID reporting willingness to use injectable PrEP. Given a choice to use oral or injectable PrEP, 56.3% of PWID preferred to use oral PrEP, 31.1% preferred an injectable PrEP, and 12.5% expressed no preference.

Reasons cited for unwillingness to use oral PrEP (Table 2) included approximately one-third of PWID with perceived a low-risk of acquiring HIV and smaller percentages noting concern about side-effects, other people thinking that they have HIV / AIDS, and reservations about possible costs. Reasons cited by PWID for unwillingness to use injectable PrEP (Table 3) were similar to those listed for oral PrEP, with low perceived HIV risk being most common. Concerns about injections themselves or related pain were cited by less than 10% of PWID. Unweighted estimates, for reasons for unwillingness to use oral and injectable PrEP tracked similarly to the estimates in unweighted analysis and they are shown in S3 & S4 Tables.

**Table 2:** Reasons participants identified for being unwilling to use *oral* pre-exposure prophylaxis among people who inject drugs and men who have sex with men in India.

****Reason****^1^	****PWID (N = 4,269, pooled %**^**2**^**)****	****MSM (N = 3,690, pooled %****^2^****)****
Side effects	18.1	63.2
Worry it won’t work	7.0	20.8
Diet and sleep might be interrupted	2.7	9.2
Drug resistance might develop	4.3	8.9
People might think I have HIV/AIDS	19.9	19.3
Cost	7.2	8.8
Hassle to take a pill every day	7.7	19.2
Not at risk for HIV	36.1	32.1

PWID: people who inject drugs; MSM: men who have sex with men.

^1^ Participants could choose more than one reason.

^2^ Trial sites pooled by stratum (12 PWID sites and 10 MSM sites) using composite weight incorporating both the RDS-II weight and a weight for population size.

**Table 3:** Reasons participants identified for being unwilling to use *injectable* pre-exposure prophylaxis among people who inject drugs and men who have sex with men in India.

****Reason****^1^	****PWID (N = 4,822, pooled %****^2^****)****	****MSM (N = 3683, pooled %****^2^****)****
Pain from injection	7.3	49.6
Side effects (other than pain from injection)	12.1	37.0
Worry it won’t work	6.5	9.9
Diet and sleep might be interrupted	3.4	5.4
Drug resistance might develop	6.6	7.4
People might think I have HIV/AIDS	19.1	13.8
Cost	7.5	7.4
Hassle to get injections	9.6	13.1
Not at risk for HIV	35.0	28.0
Do not like getting injections	2.9	15.6

PWID, people who inject drugs; MSM, men who have sex with men.

^1^Participants could choose more than one reason.

^2^Trial sites pooled by stratum (12 PWID sites and 10 MSM sites) using composite weight incorporating both the RDS-II weight and a weight for population size.

We compared characteristics in PWID groups according to willingness to use PrEP and, among those not willing to use PrEP, according to whether or not they endorsed low self-perceived HIV risk as a reason for unwillingness (Table 4; unweighted estimates shown in S5 Table). There was a tendency for participants who cited low perceived HIV risk as a reason for unwillingness to use PrEP to report lower risk behaviors. For example, 18.6% in this group reported needle/syringe sharing in the prior 6 months, compared with 24.4% who did not cite low perceived HIV risk (but were still unwilling to use PrEP) and 35.4% among those willing to use PrEP. HIV positivity, which represented either unawareness or non-disclosure of status at study entry, was substantial in all groups (range 8.5% to 14.5%)

**Table 4:** Characteristics and reported risk behaviors among PWID according to willingness to use pre-exposure prophylaxis and self-perceived risk of HIV.

****Characteristic (n, col % or median, IQR)****	****Willing to use PrEP (n = 5831, 53.66%) n, %****	****Unwilling to use PrEP****	
		Does Not Endorse self-perceived HIV risk as reason for unwillingness (n = 2388, 29.77%) n, %	Endorses a lack of self-perceived HIV risk as reason for unwillingness (n = 1617, 16.66%) n, %
****Median age****	28 (23-35)	30 (25-38)	31(25-38)
****Sex****			
Male	5548, 95.1%	2281, 95.5%	1583, 97.9%
Female	283, 4.9%	107, 4.5%	34, 2.1%
****Marital Status****			
Never married	2906, 49.8%	982, 41.1%	713, 44.1%
Married/ living with partner/ long-term relationship	2402, 41.2%	1120, 46.9%	741, 45.8%
Widowed/ divorced/ separated	522, 9.0%	285, 11.9%	163, 10.1%
****Education****			
Primary school or less	2025, 34.7%	893, 37.4%	608, 37.6%
Secondary school or beyond	3806, 65.3%	1495, 62.6%	1009, 62.4%
****Household monthly income, tertiles (INR)****			
0-10,000	2119. 36.3%	1059, 44.3%	648, 40.1%
>10,000–25,000	2181, 37.4%	878, 36.8%	660, 40.8%
> 25,000	1530, 26.2%	452, 18.9%	309, 19.1%
****Injection in prior 6 months****			
None	1258, 21.6%	681, 28.5%	476, 29.4%
Less than daily	1527, 26.2%	780, 32.7%	510, 31.5%
Daily	3046, 52.2%	927, 38.8%	631, 39.1%
****Shared needle/syringe in prior 6 months****	2066, 35.4%	582, 24.4%	300, 18.6%
****Recent HIV-positive injection or sex partner****	87, 1.5%	37, 1.5%	13, 0.8%
****Number of sex partners in prior 6 months****			
None	2658, 45.6%	962, 40.3%	764, 47.2%
One	2456, 42.1%	1111, 46.5%	690, 42.6%
Two or more	717, 12.3%	316, 13.2%	163, 10.1%
****Unprotected sex in prior 6 months****	2716, 46.6%	1299, 54.4%	741, 45.8%
****HIV prevalence****	847, 14.5%	268, 11.2%	137, 8.5%

PWID, people who inject drugs; PrEP, pre-exposure prophylaxis; INR, Indian rupees (exchange rate INR 72: USD 1).

Participant-level correlates of willingness to use PrEP among PWID are presented in Table 5. In multivariate models, sharing needles/syringes in the prior 6 months and hazardous alcohol use were significantly associated with higher odds of willingness to use PrEP, while being separated, widowed or divorced was associated with a significantly lower odds of willingness to use PrEP (relative to never being married) in weighted analysis. In general, the variance in the unweighted analyses (sensitivity) was smaller than that in the weighted analyses (primary), resulting in a larger number significant correlates in the unweighted analyses. Significant correlates of willingness to use PrEP from unweighted analyses included having sexual partners, sharing needles/syringes, unprotected sex, sex work in the prior 6 months, hazardous alcohol use, syringe service program use in prior 6 months, a higher stigma score, and incarceration in the prior 6 months (S7 Table).

**Table 5:** Correlates of willingness to use oral pre-exposure prophylaxis among people who inject drugs (PWID) in India.

****Correlate****	****Unadjusted Odds Ratio (95% CI)****^1^	****P value****	****Adjusted Odds Ratio (95% CI)****^4^
Age (per 5-year increase)	0.99 (0.95-1.04)	0.795	1.00 (0.94-1.06)
Sex			
Male	Reference		Reference
Female	1.16 (0.78-1.73)	0.468	1.2 (0.79-1.86)
Marital Status			
Never married	Reference		Reference
Married/ living with partner/ long-term relationship	1.09 (0.93-1.27)	0.292	1.08 (0.83-1.41)
Widowed/ divorced/ separated	0.79 (0.68-0.92)	0.002	0.76 (0.63-0.92)
Education			
Primary school or less	Reference		Reference
Secondary school or beyond	0.93 (0.79-1.09)	0.384	0.94(0.8-1.11)
Household monthly income, tertiles (INR)			
0-10,000 (USD 0-139)	Reference		Reference
>10,000–25,000 (USD 139-347)	0.89 (0.77-1.04)	0.150	0.89(0.76-1.05)
> 25,000 (USD 347)	0.91 (0.80-1.03)	0.140	0.92(0.8-1.07)
Number of sex partners in prior 6 months			
None	Reference		
One	1.19 (0.99-1.42)	0.062	
Two or more	1.36 (0.97-1.90)	0.075	
Recent HIV-positive injection or sex partner	0.97 (0.67-1.40)	0.855	
Ever sex with a man (men only)	0.93 (0.72-1.20)	0.571	
Injection in prior 6 months			
None	Reference		
Less than daily	0.89 (0.63-1.26)	0.505	
Daily	1.24 (0.88-1.75)	0.211	
Shared needle/syringe in prior 6 months	1.56 (1.07-2.27)	0.020	1.54(1.06-2.23)
Hazardous alcohol use^2^	1.37 (1.01-1.86)	0.043	1.37(1.01-2.23)
Unprotected sex in prior 6 months	1.19 (0.94-1.50)	0.144	
Sex work in prior 6 months	1.31 (0.93-1.83)	0.123	
Syringe service program use in prior 6 months	1.18 (0.90-1.55)	0.234	
Opioid agonist treatment in prior 6 months	1.13 (0.81-1.57)	0.477	
HIV test in prior 12 months	1.13 (0.97-1.32)	0.118	
Composite PWID stigma score (per 1-unit increase)^3^	1.02 (0.95-1.09)	0.573	
Incarcerated in prior 6 months	1.44 (0.97-2.14)	0.068	

CI, confidence interval; INR, Indian rupees (exchange rate INR 72: USD 1); PWID, people who inject drugs.

^1^Multi-level logistic model with random intercept for site and scaled RDS-II weights as probability weights.

^2^Hazardous alcohol use defined as an Alcohol Use Disorders Identification Test (AUDIT) score ≥8.

^3^Injection drug use stigma calculated as the sum of four stigma sub-scales: experienced, vicarious, community and self-stigma with each sub-scale equally weighted. Stigma scores range from 0 to 20, with higher scores indicating higher levels of stigma.

^4^ Multivariate models for PWID, in which we included demographic characteristics (age, sex, education, marital status, income) in addition to variables the were significantly (P <0.05) associated with willingness to use PrEP in univariable weighted analysis.

### Awareness and willingness to use PrEP among MSM

In analyses of data pooled across cities, awareness of PrEP was low among MSM (8.0%, 95% CI: 7.7, 8.4) with more than 10% awareness observed in three MSM cities, (Coimbatore [41%], Belgaum [18%], and Bangalore [13%]. Despite low awareness, a substantial percentage of MSM expressed willingness to use oral PrEP. In analyses of data pooled across sites, 67.6% [95% CI: 66.8, 68.4] of MSM were willing to use oral PrEP with substantial heterogeneity observed across cities. (Fig 1B; range: 33.5%, 95.4%). Willingness to use oral PrEP at the city-level was not significantly associated with city-level of PrEP awareness among MSM (ρ = -0.13, P = 0.71). Similar to PWID, there was no evidence that site allocation to the intervention or the control condition was associated with PrEP awareness or willingness to use PrEP (data not shown). Unweighted estimates of awareness and willingness to use PrEP shown in S2 Table.

Willingness to use an injectable formulation of PrEP tracked similarly with the data for oral PrEP, with 67.8% (95% CI: 67.0, 68.6) of MSM reporting willingness to use injectable PrEP. Given a choice to use oral or injectable PrEP, 39.6% of MSM preferred oral PrEP, 41.7% preferred injectable PrEP, and 18.7% expressed no preference.

Reasons cited by MSM for being unwilling to use oral and injectable PrEP are summarized in Tables 2 and 3, respectively (unweighted estimates in S3 and S4 Tables). More than half the respondents were worried about side-effects of the medications with nearly a third of the respondents perceiving a low risk of acquiring HIV.

We compared key characteristics among MSM according to willingness to use PrEP and the role of self-perceived HIV risk among those unwilling to use PrEP (Table 6; unweighted estimates in S6 Table). The distribution of established HIV risk factors among MSM - including Kothi (feminine) sexual identity, five or more partners in prior 6 months, unprotected anal intercourse in the prior 6 months, and sex work in the prior 6 months – were not notably different across these groups. Similarly, HIV-positivity by study testing, which represents either unawareness or non-disclosure of HIV status in the survey, ranged from 3.1% to 4.3% across groups.

**Table 6:** Characteristics and reported risk behaviors among MSM according to willingness to use pre-exposure prophylaxis and self-perceived risk of HIV.

****Characteristic (n, col % or median, IQR)****	****Willing to use PrEP (n = 5278, 67.6%) n, %****	****Unwilling to use PrEP****	
		Does not endorse self-perceived HIV risk as reason for unwillingness (n = 2167, 22.5%)	Endorses a lack of self-perceived HIV risk as reason for unwillingness (n = 1069, 9.9%)
****Median age****	27 (22-35)	27 (22-35)	28 (22-37)
****Marital Status****			
Never married	n = 2508, 47.5%	n = 1205, 55.6%	n = 474, 44.3%
Married/ living with partner/ long-term relationship	2608, 49.4%	n = 895, 41.3%	n = 547, 51.1%
Widowed/ divorced/ separated	162, 3.1%	n = 67, 3.1%	n = 48, 4.5%
****Sexual identity****			
Panthi	2273, 43.1%	n = 866, 40.0%	n = 432, 40.4%
Kothi	937, 17.8%	n = 471, 21.7%	n = 200, 18.7%
Double-Decker	1230, 23.3%	660, 30.5%	288, 27.0%
Gay/MSM	95, 1.8%	14, 0.7%	13, 1.2%
Bisexual	743, 14.1%	155, 7.1%	136, 12.8%
****Education****			
Primary school or less	764, 14.5%	336, 15.5%	199, 18.6%
Secondary school or beyond	4514, 85.5%	1831, 84.5%	870, 81.4%
****Household income, tertiles (INR)****			
0-11,000	1576, 29.9%	623, 28.8%	363, 33.9%
>11,000-20,000	2144, 40.6%	935, 43.2%	389, 36.7%
> 20,000	1558, 29.5%	608, 28.1%	318, 29.7%
****Number of male partners in prior 6 months****			
None or one	2532, 48.0%	1077, 49.7%	500, 46.8%
Two to four	1722, 32.6%	703, 32.4%	320, 30.0%
Five or more	1024, 19.4%	387, 17.96%	249, 23.3%
****Main male partner****	4398, 83.3%	1474, 68.0%	863, 80.7%
****Type of anal sex with last 4 partners****			
No anal sex	923, 17.5%	362, 16.7%	193, 18.0%
Only penetrative	2046, 38.8%	833, 38.4%	454, 42.4%
Receptive (only or both penetrative and receptive)	2309, 43.6%	973, 44.9%	423, 39.5%
****Unprotected anal intercourse in prior 6 months****	2482, 47.0%	1188, 54.8%	520, 48.7%
****Sex work in prior 6 months****	832, 15.8%	438, 20.2%	243, 22.7%
****Recent HIV-positive sex or injecting partner****	101, 1.9%	21, 1.0%	10, 0.9%
****Symptoms of STI in prior 6 months****	235, 4.5%	70, 3.24%	27, 2.6%
****Active syphilis infection****	335, 6.4%	142, 6.6%	55, 5.1%
****HSV-2 positive****	1004, 19.0%	406, 18.8%	193, 18.1%
****Injected drugs in prior 6 months****	30, 0.6%	7, 0.3%	1, 0.1%
****HIV prevalence****	228, 4.3%	70, 3.2%	33, 3.1%

MSM, men who have sex with men; PrEP, pre-exposure prophylaxis; INR, Indian rupees (exchange rate INR 72: USD 1).

Participant-level correlates of willingness to use oral PrEP in MSM are presented in Table 7. In multivariate models using sampling weights, having a main male sexual partner and injection drug use in the prior 6 months were significantly associated with higher odds of willingness to use PrEP. As we observed for the PWID, the variance in the unweighted analyses (sensitivity) was smaller than that in the weighted analyses (primary), resulting in a larger number of significant correlates in the unweighted analyses. Among MSM in unweighted analyses, identifying as *kothi* (feminine) or gay/MSM, having five or more sexual partners in the prior 6 months, having a male as main sexual partner, symptoms of a sexually transmitted infection in the prior 6 months, hazardous alcohol use, HIV testing in the prior 12 months, and higher stigma scores were significantly associated with willingness to use PrEP (S8 Table).

**Table 7:** Correlates of willingness to use oral pre-exposure prophylaxis among men who have sex with men (MSM) in India.

****Correlate****	****Unadjusted Odds Ratio (95% CI)****^1^	****P value****	****Adjusted Odds Ratio (95% CI)****^5^
Age (per 5-year increase)	1.01 (0.97-1.06)	0.616	1,01 (0.94-1.08)
Marital Status			
Never married	Reference		Reference
Married/ living with partner/ long-term relationship	1.07 (0.82-1.41)	0.618	1.01 (0.70-1.47)
Widowed/ divorced/ separated	0.91 (0.66-1.26)	0.588	0.84 (0.56-1.28)
Sexual identity			
Panthi^2^	Reference		Reference
Kothi^2^	0.84 (0.56-1.26)	0.398	0.84 (0.56-1.26)
Double-Decker	0.92 (0.65-1.31)	0.644	0.92 (0.65-1.28)
Gay/MSM	1.17 (0.41-3.33)	0.767	1.2 (0.45-3.44)
Bisexual	1.13 (0.81-1.60)	0.470	1.29 (0.83-1.24)
Education			
Primary school or less	Reference		Reference
Secondary school or beyond	1.01 (0.83-1.23)	0.919	1.02 (0.83-1.24)
Household income, tertiles (INR)			
0-11,000 (USD 0-153)	Reference		Reference
>11,000-20,000 (USD 153-278)	1.02 (0.80-1.29)	0.903	0.99(0.78-1.25)
> 20,000 (USD >278)	0.93 (0.66-1.31)	0.682	0.89(0.62-1.29)
Number of male partners in prior 6 months			
None or One	Reference		
Two to four	0.96 (0.72-1.27)	0.759	--
Five or more	1.16 (0.75-1.78)	0.503	--
Main male partner	2.33 (1.22-4.45)	0.010	2.3 (1.28-4.45)
Type of anal sex with last 4 partners			
No anal sex	Reference		
Only penetrative	0.89 (0.53-1.51)	0.675	--
Receptive (only or both penetrative and receptive)	0.91 (0.53-1.54)	0.719	--
Unprotected anal intercourse in prior 6 months	1.00 (0.63-1.57)	0.988	--
Sex work in prior 6 months	0.84 (0.49-1.43)	0.527	--
Recent HIV-positive sex or injecting partner	1.07 (0.67-1.69)	0.785	--
Symptoms of STI in prior 6 months	1.62 (0.73-3.62)	0.235	--
Syphilis positive	0.99 (0.75-1.29)	0.913	--
HSV-2 positive	1.01 (0.83-1.23)	0.914	--
Hazardous alcohol use^3^	1.04 (0.64-1.69)	0.877	--
Injected drugs in prior 6 months	2.67 (1.89-3.77)	<0.001	2.5 (1.74-3.64)
HIV test in prior 12 months	1.10 (0.80-1.53)	0.554	--
Composite MSM stigma score (per 1-unit increase)^4^	0.99 (0.87-1.13)	0.882	--

CI, confidence interval; INR, Indian rupees (exchange rate INR 72: USD 1); STI, sexually transmitted infection; HSV-2, herpes simplex virus type-2.

^1^ Multi-level logistic model with random intercept for site and scaled RDS-II weights as probability weights.

^2^ Panthi and kothi refer to masculine and feminine sexual identities, respectively.

^3^ Hazardous alcohol use defined as an Alcohol Use Disorders Identification Test (AUDIT) score ≥8.

^4^ MSM stigma calculated as the sum of four stigma sub-scales: experienced, vicarious, community and self-stigma with each sub-scale equally weighted. Stigma scores range from 0 to 20, with higher scores indicating higher levels of stigma.

^5^ Multivariate models for MSM, in which we included demographic characteristics (age, sex, sexual identity, education, marital status, income) in addition to variables the were significantly (P <0.05) associated with willingness to use PrEP in univariable weighted analysis.

## Discussion

As in most countries outside of sub-Saharan Africa, the burden of HIV is borne disproportionately by key populations in India [25]. In the absence of an effective vaccine, there is a need to optimize a multi-modality prevention approach that includes PrEP to prevent HIV acquisition. The World Health Organization has increasingly advocated accelerating implementation of PrEP as part of HIV combination prevention in low- to middle-income countries. In India, although available in the private sector since late 2016, PrEP has not been rolled out as part of a public sector National AIDS Control Program. However, a new national policy is in the pipeline, adding to the relevance of the data presented here.

In this multi-city, community-based survey, we found very low PrEP awareness (less than 10%) among PWID and MSM. However, following a brief description of oral PrEP, over half of PWID and MSM reported willingness to use PrEP, with a higher percentage of MSM than PWID expressing willingness. Our results match similar findings of low awareness but higher willingness to use PrEP among MSM and transgender individuals that have been published in India and other countries in the region, such as Myanmar, Vietnam and Thailand [16, 17, 26–30] There is a paucity of data on awareness and willingness to use PrEP among PWID, particularly in low- or middle-income countries, which makes our study, including two key populations, unique. The wide variability in willingness to use PrEP (for both groups) across different cities was notable. In site-level analyses, willingness was associated with neither PrEP awareness nor HIV prevalence among the key populations, indicating implementation of a PrEP roll-out likely cannot be a one-size-fits-all for different cities and communities across India.

Among both PWID and MSM who said they would not be willing to use PrEP, the most common reason cited for unwillingness was a low self-perceived risk for HIV. Participants who endorsed low HIV risk as a reason for unwillingness to use PrEP tended to report fewer HIV risk behaviors than those who did not cite low HIV risk as a reason against using PrEP or those who were willing to use PrEP. However, the differences across these groups were often small, particularly among MSM. Perceptions of low-HIV risk, side-effects from PrEP, and concerns about being labeled as HIV-positive are commonly identified barriers to PrEP [31], which underscores the need for ongoing HIV education and strategies to reduce HIV-related stigma before and during implementation of PrEP in these communities to ensure PrEP demand. In primary weighted analyses, PWID with higher risk behaviors - needle/syringe sharing and hazardous alcohol use - were more likely to be willing to use PrEP which suggests that PWID that would benefit most would also the most likely to use it. However in unweighted analyses, additional correlates of willingness to use PrEP included having sexual partners, unprotected sex, sex work in the prior 6 months, syringe service program use in prior 6 months, a higher stigma score, and incarceration in the prior 6 months In the primary weighted analyses for MSM, having a main male partner and recent injection drug use (which was reported by less than 1% but is also a very high-risk sub-group [6]) were the only correlates of PrEP willingness; however, in the unweighted sensitivity analysis, additional established HIV risk behaviors were identified as significant correlates; a *kothi* (feminine) or gay/MSM sexual identity, a larger number of partners, having a male as main sexual partner, recent symptoms of a sexually transmitted infection, hazardous alcohol use and higher stigma score.

These findings have implications for scaling up public sector PrEP in India. First, and not unexpectedly, awareness of PrEP was low in these key populations in a setting where there has been minimal efforts to disseminate information about or provide free access to PrEP. A multi-stage and key population-targeted education campaign will be needed to increase PrEP literacy and generate demand. Second, lack of knowledge about HIV and underestimation of personal HIV risk remain barriers to the uptake of PrEP and other HIV prevention interventions. In this regard, bundling PrEP with key populations services (e.g., opioid agonist treatment or sexually transmitted infection services) may expedite linking individuals at highest risk to PrEP and mitigate risk compensation [32, 33]. In unweighted analyses, we found that use of syringe service programs among PWID and recent HIV testing among MSM were significantly associated with increased willingness to use PrEP. This highlights the potential of using exiting service modalities to deliver PrEP. Third, although over half of PWID and MSM expressed willingness to use PrEP, it is important to acknowledge that PrEP roll-out and reaching the highest risk individuals has been challenging even in high-income countries [34]. Consequently, education is likely to be insufficient to get PrEP to those likely to benefit. Fourth, 49.0% and 67.8% of PWID and MSM, respectively, expressed willingness to use injectable PrEP, which has been found to be more effective than oral PrEP [35]. End users of PrEP could benefit if given a choice of using an oral or an injectable PrEP, considering that injectables could decrease concerns about adherence [36]. Achieving significant uptake of a new prevention method is a complex undertaking that may differ by region and involve an interplay of individual and, family, social, religious and cultural factors [11].

This study has strengths that include a large community-based sample of PWID and MSM across multiple regions and cities in India and detailed information on individuals’ willingness and beliefs surrounding PrEP use. The study also has limitations. First, cities were not selected in a way to produce nationally representative data for PWID or MSM. Second, the population surveys were conducted as the final phase of a cluster-randomized trial of a key population-focused structural intervention. However, the intervention did not include PrEP or education about PrEP [3], and there was no evidence that site allocation to the intervention or the control condition was associated with PrEP awareness or willingness to use PrEP. Third, there could be changes in the levels of awareness and willingness to use PrEP since data was collected in 2016-17. Fourth, when we asked about PrEP awareness, we only used the term “pre-exposure prophylaxis” but later, when we ask about willingness to use PrEP, we provide alternative descriptions as described. This could have led to underestimation of the awareness levels.

## Conclusion

In conclusion, prior to public sector implementation of PrEP, we found very low awareness of this prevention modality among PWID and MSM surveyed across multiple regions of India. Although larger fractions expressed willingness to use PrEP, low self-perceived HIV risk and concerns about side effects and being identified as HIV-positive will likely be barriers to uptake. There was modest evidence that high-risk behaviors were associated with increased willingness to use PrEP among both groups. These data highlight opportunities and challenges for PrEP rollout in India and other low- or middle-income countries.

## Supporting information

S1 Text Survey questions addressing PrEP. (PDF)

S1 Fig Awareness about oral PrEP in 12 PWID sites (a) and in 10 MSM sites (b).
(PDF)

S1 Table Characteristics of people who inject drugs and men who have sex with men who participated in a respondent-driven sampling survey across 22 sites in India between August 2016 and May 2017. (DOCX)

S2 Table Awareness of and willingness to use oral pre-exposure prophylaxis among PWID and MSM in India by site, unweighted. (DOCX)

S3 Table Reasons participants identified for being unwilling to use oral pre-exposure prophylaxis among PWID and MSM in India, unweighted. (DOCX)

S4 Table Reasons participants identified for being unwilling to use injectable pre-exposure prophylaxis among PWID and MSM in India, unweighted. (DOCX)

S5 Table Characteristics and reported risk behaviors among PWID according to willingness to use pre-exposure prophylaxis and self-perceived risk of HIV, unweighted estimates. (DOCX)

S6 Table Characteristics and reported risk behaviors among MSM according to willingness to use pre-exposure prophylaxis and self-perceived risk of HIV, unweighted analysis. (DOCX)

S7 Table Correlates of willingness to use oral pre-exposure prophylaxis among people who inject drugs (PWID) in India, unweighted. (DOCX)

S8 Table Correlates of willingness to use oral pre-exposure prophylaxis among men who have sex with men (MSM) in India, unweighted. (DOCX)
