# Supplementary material for: Awareness of and willingness to use pre-exposure prophylaxis (PrEP) among people who inject drugs and men who have sex with men in India: Results from a multi-city cross-sectional survey
Source: PLoS One. 2021 Feb 25;16(2):e0247352. doi: 10.1371/journal.pone.0247352 (PMC7906475; doi:10.1371/journal.pone.0247352)
Supplement: S1 Text — (PDF) [file pone.0247352.s001.pdf]

S1 Text

Survey questions addressing PrEP

Have you heard of the following as ways to prevent HIV infection? **(SELECT ALL THAT APPLY**)

	Yes	No
1a. Male Circumcision (Khatna)	1	0
1b. Vaginal/Rectal Microbicides	1	0
1c. Pre-Exposure Prophylaxis	1	0
1d. Treatment as Prevention	1	0
1e. No to all of the above options	1	0

2. There is now evidence that people who are not infected with HIV could take an HIV treatment pill daily to reduce their risk of becoming infected. We call these medicines PrEP. Would you be willing to take PrEP every day to reduce your risk of getting infected with HIV?

- ☐ No chance
- ☐ Very little chance
- ☐ Some chance
- ☐ Very good chance
- ☐ Undecided

3a. Why would you not be willing to take an HIV treatment pill every day? (**Select All That Apply**)

	Yes	No
3a1. Side effects	1	0
3a2. Worry that the treatment won’t work	1	0
3a3. Diet and sleep might be interrupted	1	0
3a4. Drug resistance might develop	1	0
3a5. People might think I have HIV/AIDS	1	0
3a6. Cost	1	0
3a7. It is a hassle to take a pill every day	1	0
3a8. I am not at risk for HIV	1	0
3a9. Other	1	0
3a10. Don’t Know	1	0
3a11. Refused	1	0

3b. What if these PrEP medicines could be given to HIV uninfected individuals as injections every 1 to 2 months to reduce their risk of becoming infected? Would you be willing to take an injection every 1 to 2 months to reduce your risk of getting infected with HIV?

- ☐ No chance
- ☐ Very little chance

- ☐ Some chance
- ☐ Very good chance
- ☐ Undecided

3c. Why would you not be willing to take an injection every 1 to 2 months? (**SELECT ALL THAT APPLY**)

	**Yes**	**No**
3c1. Pain from the injection	1	0
3c2. Side effects other than pain from the injections	1	0
3c3. Worry that the injections won't work	1	0
3c4. Diet and sleep might be interrupted	1	0
3c5. Drug resistance might develop	1	0
3c6. People might think I have HIV/AIDS	1	0
3c7. Cost	1	0
3c8. It is a hassle to get injections	1	0
3c9. I am not at risk for HIV	1	0
3c10. I do not like taking injections	1	0
3c11. Other	1	0
3c12. Don't Know	1	0
3c13. Refused	1	0

3d. If you had a choice to use a daily pill or an injection everyone to two months to protect yourself from HIV, which would you choose?

- ☐ Prefer the pill
- ☐ Prefer the injection/shot
- ☐ I would feel the same about taking either pills or injections

3e: Why would you prefer the injection/shot to the pills? (**SELECT ALL THAT APPLY**)

		**Yes**	**No**
3e1.	An injection is less of a hassle	1	0
3e2.	An injection does not require a daily reminder like pills	1	0
3e3.	I do not like having to carry pills/pill bottle with me	1	0
3e4.	I do not like swallowing pills	1	0
3e5.	I do not like people seeing me take pills	1	0
3e6.	An injection requires fewer doctor or pharmacist visits	1	0
3e7.	Injections work better than pills	1	0
3e8.	Other	1	0
3e9.	Don't Know	1	0
3e10.	Refused	1	0
