# Supplementary figures and images for: Awareness of and willingness to use pre-exposure prophylaxis (PrEP) among people who inject drugs and men who have sex with men in India: Results from a multi-city cross-sectional survey
Source: PLoS One. 2021 Feb 25;16(2):e0247352. doi: 10.1371/journal.pone.0247352 (PMC7906475; doi:10.1371/journal.pone.0247352)

**Supplemental Figure 1: Awareness about oral PrEP in 12 PWID sites (a) and in 10 MSM sites (b)**

**(a)**

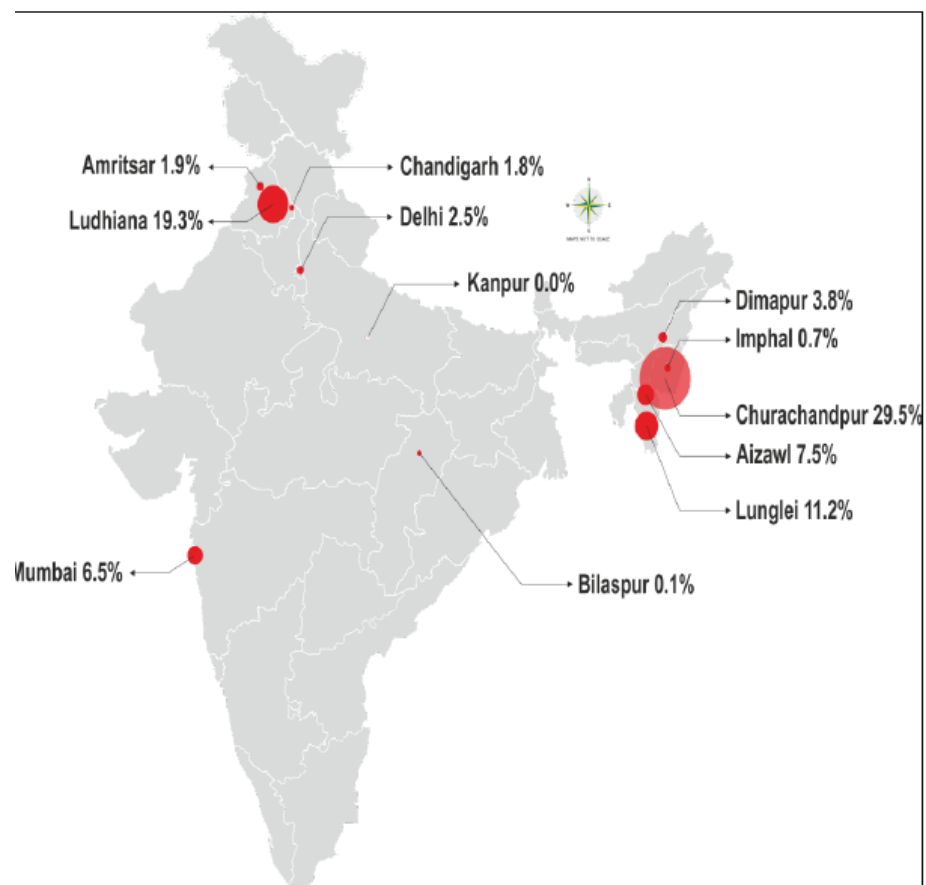

**(b)**

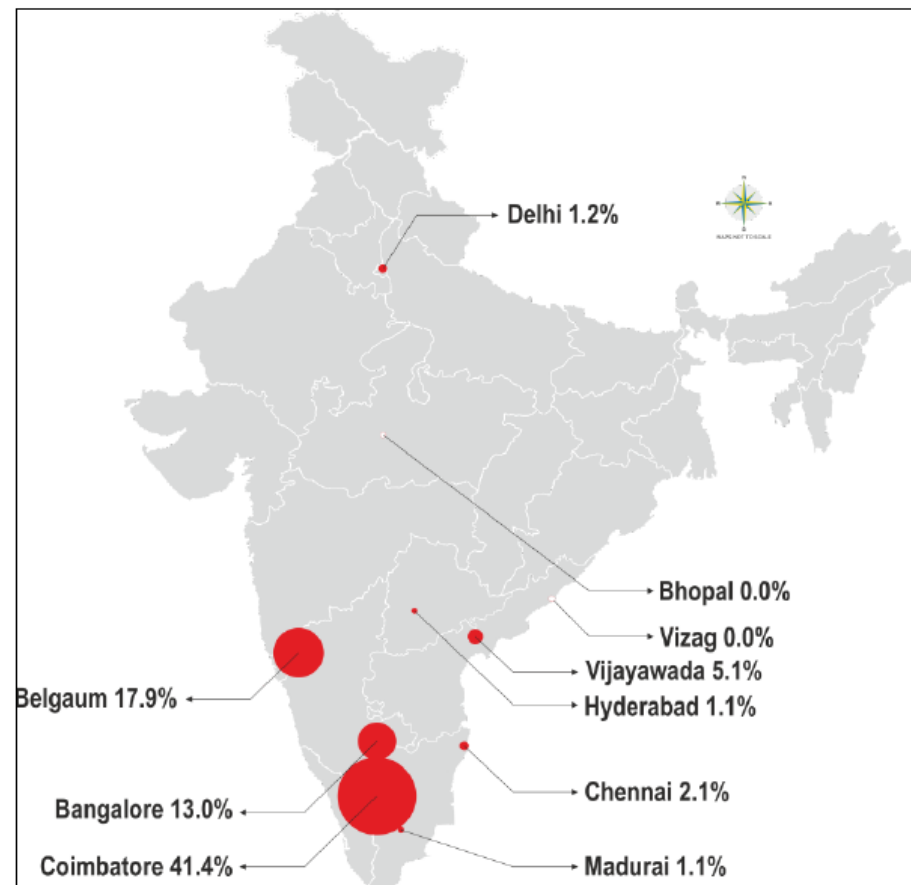

Supplement: S1 Fig — Awareness about oral PrEP in 12 PWID sites (a) and in 10 MSM sites (b). (PDF) [file pone.0247352.s002.pdf]
