# Supplementary material for: Awareness of and willingness to use pre-exposure prophylaxis (PrEP) among people who inject drugs and men who have sex with men in India: Results from a multi-city cross-sectional survey
Source: PLoS One. 2021 Feb 25;16(2):e0247352. doi: 10.1371/journal.pone.0247352 (PMC7906475; doi:10.1371/journal.pone.0247352)
Supplement: S1 Table — (DOCX) [file pone.0247352.s003.docx]

**S1 Table. Characteristics of people who inject drugs and men who have sex with men who participated in a respondent-driven sampling survey across 22 sites in India between August 2016 and May 2017.**

| **n individuals, median % (range)^1^** | **PWID sites (N=12 sites, N=10538 individuals)** | **MSM sites (N=10 sites, N=8621 individuals)** |
| --- | --- | --- |
| Median age | 28 (25-35) | 28 (22-31) |
| Female | 333, 1.4 (0.1-12.5) | -- |
| MSM identity  *Panthi*^2^  *Kothi*^2^  *Double-decker*^2^  MSM/gay^2^  Bisexual^2^ | -- | 3307, 37.6 (7.8-82.5)  2051, 23.2 (6.4-50.2)  2354, 30.0 (6.8-44.0)  155, 1.1 (0-6.3)  750, 3.1 (0-27.8) |
| Marital status  Never married  Married/living with partner/long-term partner  Widowed/divorced/separated | 5232, 49.5 (33.0-63.4)  4125, 40.5 (21.1-59.2)  1181, 9.6 (2.0-28.8) | 4016, 54.5 (10.8-70.1)  4340, 40.6 (27.7-88.7)  265, 2.2 (0.5-8.5) |
| Completed secondary school | 7239, 70.1 (35.8-97.1) | 7095, 84.8 (71.2-89.5) |
| Median household median income (INR) | 15,000 (10,000-50,000) | 15,000 (12,000-20,000) |
| Incarceration in prior 6 months | 983, 6.7 (4.2-25.6) | 276, 2.1 (0.1-14.8) |
| Hazardous alcohol use^3^ | 3901, 35.9 (9.7-55.8) | 2839, 39.3 (16.4-47.5) |
| Injected drugs in prior 6 months | 8572, 87.8 (31.6-99.0) | 48, 0.3 (0-2.6) |
| Shared needle/syringe in prior 6 months | 3647, 37.3 (3.2-70.3) | 0 |
| NSEP participation in prior 6 months | 3039, 27.2 (2.9-73.1) | -- |
| OAT in prior 6 months | 2733, 30.2 (1.6-52.0) | -- |
| Median number of sexual partners^4^ in prior 6 months | 1 (0-1) | 2 (1-6) |
| Unprotected sex^5^ in prior 6 months | 4990, 48.1 (22.7-77.9) | 4542, 52.3 (27.8-71.1) |
| Sex work in prior 6 months | 359, 2.2 (0.2-11.0) | 2359, 23.9 (17.4-46.2) |
| Active syphilis infection ^6^ | -- | 582, 6.3 (3.1-11.2) |
| HSV-2-infected^6^ | -- | 1710, 27.1 (20.3-38.3) |
| HIV test in prior 12 months^6^ | 3534, 34.5 (3.1-56.5) | 2652, 33.1 (14.8-43.6) |
| HIV-infected^6^ | 1400, 10.4 (3.9-35.6) | 401, 4.4 (1.8-11.2) |
| Median drug use/MSM behavior-related stigma^7^ | 7.6 (3.3-11.1) | 4.1 (1.6-6.7) |

PWID, people who inject drugs; MSM, men who have sex with men; HSV-2, herpes simplex virus type 2. INR, Indian rupees (exchange rate INR 72: USD 1)

^1^Data shown as percent at the median site (site range), unless otherwise noted. Estimates are unweighted.

^2^*Panthi* and *kothi* refer to masculine and feminine sexual identities, respectively. Men who self-identify as panthi tend to have masculine mannerisms/ appearance and may mainly or only engage in penetrative intercourse with men. Men who self-identify as kothi often show more feminine mannerisms/ appearance and mainly or only engage in receptive anal intercourse with other men. Men who self-identify as Double-decker may have feminine or masculine mannerisms/appearance and engage in both receptive and penetrative intercourse with men. Men who self-identify as gay, bisexual or MSM have fluid identities / behaviors that change over time

^3^Hazardous alcohol use defined as an Alcohol Use Disorders Identification Test (AUDIT) score ≥8.

^4^Male and female sexual partners for PWID, male sexual partners for MSM

^5^Vaginal or anal sex for PWID, anal sex for MSM

^6^Composite stigma score including sub-scales: enacted, vicarious, felt normative, and internalized stigma; range: 0-20, with higher scores indicating more stigma. The scale had accepted reliability with Cronbach’s α=0.90 for PWID and 0.96 for MSM)

^7^According to serum testing done at the survey visit.
