# Supplementary material for: Awareness of and willingness to use pre-exposure prophylaxis (PrEP) among people who inject drugs and men who have sex with men in India: Results from a multi-city cross-sectional survey
Source: PLoS One. 2021 Feb 25;16(2):e0247352. doi: 10.1371/journal.pone.0247352 (PMC7906475; doi:10.1371/journal.pone.0247352)
Supplement: S2 Table — (DOCX) [file pone.0247352.s004.docx]

**S2 Table:** Awareness of and willingness to use oral pre-exposure prophylaxis among people who inject drugs (PWID) and men who have sex with men (MSM) in India by site, **unweighted**

|  | **Aware, n (%)** | **Willing to use, n (%)** |
| --- | --- | --- |
| **PWID** |  |  |
| Aizawl | 59 (6.3) | 745 (79.9) |
| Amritsar | 21 (2.3) | 572 (65.0) |
| Bilaspur | 3 (0.3) | 528 (58.3) |
| Chandigarh | 18 (1.9) | 766 (81.2) |
| Churachandpur | 217 (25.3) | 557 (64.9) |
| Delhi | 24 (2.6) | 721 (90.0) |
| Dimapur | 40 (4.4) | 328 (36.5) |
| Imphal | 13 (1.7) | 325 (46.8) |
| Kanpur | 0 (0) | 147 (16.1) |
| Ludhiana | 160 (18.2) | 389 (73.4) |
| Lunglei | 104 (11.5) | 379 (46.3) |
| Mumbai | 41 (6.1) | 374 (56.8) |
|  |  |  |
| **MSM** |  |  |
| Bengaluru | 144 (16.1) | 586 (65.8) |
| Belgaum | 96 (10.4) | 564 (66.7) |
| Bhopal | 0 (0) | 531 (60.3) |
| Chennai | 35 (3.6) | 508 (53.1) |
| Coimbatore | 290 (32.6) | 509 (57.4) |
| Delhi | 18 (2.1) | 620 (73.4) |
| Hyderabad | 17 (2.0) | 422 (50.1) |
| Madurai | 10 (1.2) | 379 (45.7) |
| Vijayawada | 78 (10.2) | 413 (55.3) |
| Vishakhapatnam | 0 (0) | 746 (94.4) |
