# Supplementary material for: Awareness of and willingness to use pre-exposure prophylaxis (PrEP) among people who inject drugs and men who have sex with men in India: Results from a multi-city cross-sectional survey
Source: PLoS One. 2021 Feb 25;16(2):e0247352. doi: 10.1371/journal.pone.0247352 (PMC7906475; doi:10.1371/journal.pone.0247352)
Supplement: S3 Table — (DOCX) [file pone.0247352.s005.docx]

**S3 Table:** Reasons participants identified for being unwilling to use *oral* pre-exposure prophylaxis among PWID and MSM in India, **unweighted**

| **Reason^1^** | **PWID**  **(N=4,269, pooled %^2^)** | **MSM**  **(N=3,690, pooled %^2^)** |
| --- | --- | --- |
| Side effects | 19.2 | 62.0 |
| Worry it won’t work | 6.3 | 24.7 |
| Diet and sleep might be interrupted | 2.0 | 7.0 |
| Drug resistance might develop | 2.6 | 6.1 |
| People might think I have HIV/AIDS | 11.4 | 13.9 |
| Cost | 5.6 | 11.3 |
| Hassle to take a pill every day | 11.5 | 13.9 |
| Not at risk for HIV | 40.7 | 33.5 |

PWID: people who inject drugs; MSM: men who have sex with men

^1^ Participants could choose more than one reason

^2^ Pooling trial sites by stratum (12 PWID sites and 10 MSM sites)
