# Supplementary material for: Awareness of and willingness to use pre-exposure prophylaxis (PrEP) among people who inject drugs and men who have sex with men in India: Results from a multi-city cross-sectional survey
Source: PLoS One. 2021 Feb 25;16(2):e0247352. doi: 10.1371/journal.pone.0247352 (PMC7906475; doi:10.1371/journal.pone.0247352)
Supplement: S4 Table — (DOCX) [file pone.0247352.s006.docx]

**S4 Table:** Reasons participants identified for being unwilling to use *injectable* pre-exposure prophylaxis among PWID and MSM in India, **unweighted**

| **Reason^1^** | **PWID**  **(N=4,822, pooled %^2^)** | **MSM**  **(N=3683, pooled %^2^)** |
| --- | --- | --- |
| Pain from injection | 11.1 | 47.4 |
| Side effects (other than pain from injection) | 11.5 | 37.3 |
| Worry it won’t work | 6.2 | 13.1 |
| Diet and sleep might be interrupted | 2.8 | 3.3 |
| Drug resistance might develop | 4.2 | 4.1 |
| People might think I have HIV/AIDS | 10.3 | 9.3 |
| Cost | 5.6 | 9.8 |
| Hassle to get injections | 14.7 | 10.1 |
| Not at risk for HIV | 38.5 | 28.8 |
| Do not like getting injections | 4.9 | 10.4 |

PWID, people who inject drugs; MSM, men who have sex with men

1 Participants could choose more than one reason

2 Pooling trial sites by stratum (12 PWID sites and 10 MSM sites)
