# Supplementary material for: Awareness of and willingness to use pre-exposure prophylaxis (PrEP) among people who inject drugs and men who have sex with men in India: Results from a multi-city cross-sectional survey
Source: PLoS One. 2021 Feb 25;16(2):e0247352. doi: 10.1371/journal.pone.0247352 (PMC7906475; doi:10.1371/journal.pone.0247352)
Supplement: S5 Table — (DOCX) [file pone.0247352.s007.docx]

**S5 Table: Characteristics and reported risk behaviors among PWID according to willingness to use pre-exposure prophylaxis and self-perceived risk of HIV, unweighted estimates**

| **Characteristic (n, col % or median, IQR)** | **Willing to use PrEP (n=5831),  n,** | **Unwilling to use PrEP** | |
| --- | --- | --- | --- |
|  |  | Does not endorse self-perceived HIV risk as reason as reason for unwillingness  (n=2388), n | Endorses a lack of self-perceived HIV risk as reason for unwillingness  (n=1617), n |
| **Median age** | 28 (23-35) | 30 (24-36) | 30 (24-37) |
| **Sex** |  |  |  |
| Male | 5633 | 2297 | 1590 |
| Female | 195 | 91 | 27 |
| **Marital Status** |  |  |  |
| Never married | 2905 | 1154 | 771 |
| Married/ living with partner/ long-term relationship | 2258 | 945 | 672 |
| Widowed/ divorced/ separated | 668 | 289 | 174 |
| **Education** |  |  |  |
| Primary school or less | 1771 | 708 | 583 |
| Secondary school or beyond | 4060 | 1680 | 1034 |
| **Household monthly income, tertiles (INR)** |  |  |  |
| 0-10,000 | 1909 | 870 | 545 |
| >10,000 - 25,000 | 2183 | 789 | 649 |
| > 25,000 | 1739 | 729 | 423 |
| **Injection in prior 6 months** |  |  |  |
| None | 1022 | 495 | 371 |
| Less than daily | 1387 | 749 | 545 |
| Daily | 3422 | 1144 | 701 |
| **Shared needle/syringe in prior 6 months** | 2380 | 648 | 359 |
| **Recent HIV-positive injection or sex partner** | 109 | 32 | 13 |
| **Number of sex partners in prior 6 months** |  |  |  |
| None | 2614 | 1113 | 810 |
| One | 2405 | 982 | 644 |
| Two or more | 812 | 293 | 163 |
| **Unprotected sex in prior 6 months** | 2746 | 1167 | 694 |
| **HIV prevalence** | 838 | 307 | 152 |

PWID, people who inject drugs; PrEP, pre-exposure prophylaxis; INR, Indian rupees (exchange rate INR 72: USD 1). Estimates are unweighted
