# Supplementary material for: Awareness of and willingness to use pre-exposure prophylaxis (PrEP) among people who inject drugs and men who have sex with men in India: Results from a multi-city cross-sectional survey
Source: PLoS One. 2021 Feb 25;16(2):e0247352. doi: 10.1371/journal.pone.0247352 (PMC7906475; doi:10.1371/journal.pone.0247352)
Supplement: S6 Table — (DOCX) [file pone.0247352.s008.docx]

**S6 Table: Characteristics and reported risk behaviors among MSM according to willingness to use pre-exposure prophylaxis and self-perceived risk of HIV, unweighted analysis**

| **Characteristic (n, col % or median, IQR)** | **Willing to use PrEP (n=5278, 67.6%) n** | **Unwilling to use PrEP** | |
| --- | --- | --- | --- |
|  |  | Does not endorse self-perceived HIV risk as reason as reason for unwillingness  (n=2167), n | Endorses a lack of self-perceived HIV risk as reason for unwillingness  (n=1069), n |
|  |  |  |  |
|  |  |  |  |
| **Median age** | 27 (22-35) | 27 (22-35) | 29 (23-37) |
| **Marital Status** |  |  |  |
| Never married | 2348 | 1173 | 454 |
| Married/ living with partner/ long-term relationship | 2781 | 915 | 579 |
| Widowed/ divorced/ separated | 149 | 79 | 36 |
| **Sexual identity** |  |  |  |
| Panthi | 1999 | 823 | 462 |
| Kothi | 1279 | 504 | 230 |
| Double-Decker | 1341 | 680 | 293 |
| Gay/MSM | 118 | 25 | 10 |
| Bisexual | 540 | 135 | 74 |
| **Education** |  |  |  |
| Primary school or less | 889 | 390 | 213 |
| Secondary school or beyond | 4389 | 1777 | 856 |
| **Household income, tertiles (INR)** |  |  |  |
| 0-11,000 | 1770 | 660 | 425 |
| >11,000-20,000 | 2102 | 974 | 391 |
| > 20,000 | 1406 | 533 | 253 |
| **Number of male partners in prior 6 months** |  |  |  |
| None or one | 2021 | 778 | 526 |
| Two to four | 1622 | 790 | 301 |
| Five or more | 1635 | 599 | 242 |
| **Main male partner** | 4703 | 1757 | 889 |
| **Type of anal sex with last 4 partners** |  |  |  |
| No anal sex | 947 | 334 | 198 |
| Only penetrative | 1644 | 735 | 464 |
| Receptive (only or both penetrative and receptive) | 2687 | 1098 | 407 |
| **Unprotected anal intercourse in prior 6 months** | 2744 | 1221 | 523 |
| **Sex work in prior 6 months** | 1440 | 657 | 238 |
| **Recent HIV-positive sex or injecting partner** | 108 | 29 | 10 |
| **Symptoms of STI in prior 6 months** | 275 | 85 | 29 |
| **Active syphilis infection** | 372 | 148 | 61 |
| **HSV-2 positive** | 1060 | 431 | 207 |
| **Injected drugs in prior 6 months** | 35 | 10 | 3 |
| **HIV prevalence** | 270 | 89 | 39 |

MSM, men who have sex with men; PrEP, pre-exposure prophylaxis; INR, Indian rupees (exchange rate INR 72: USD 1). Estimates are unweighted
