# Supplementary material for: Awareness of and willingness to use pre-exposure prophylaxis (PrEP) among people who inject drugs and men who have sex with men in India: Results from a multi-city cross-sectional survey
Source: PLoS One. 2021 Feb 25;16(2):e0247352. doi: 10.1371/journal.pone.0247352 (PMC7906475; doi:10.1371/journal.pone.0247352)
Supplement: S7 Table — (DOCX) [file pone.0247352.s009.docx]

**S7 Table:** Correlates of willingness to use oral pre-exposure prophylaxis among people who inject drugs (PWID) in India, **unweighted**

| **Correlate** | **Unadjusted Odds Ratio**  **(95% CI)^1^** | **P-value** |
| --- | --- | --- |
| Age (per 5-year increase) | 1.00 (0.97-1.02) | 0.776 |
| Sex |  |  |
| Male | Reference |  |
| Female | 1.17 (0.91-1.50) | 0.218 |
| Marital Status |  |  |
| Never married | Reference |  |
| Married/ living with partner/ long-term relationship | 1.11 (1.01-1.23) | 0.028 |
| Widowed/ divorced/ separated | 1.02 (0.88-1.18) | 0.805 |
| Education |  |  |
| Primary school or less | Reference |  |
| Secondary school or beyond | 0.91 (0.82-1.02) | 0.102 |
| Household monthly income, tertiles (INR) |  |  |
| 0-10,000 | Reference |  |
| >10,000 - 25,000 | 0.94 (0.84-1.04) | 0.223 |
| > 25,000 | 0.85 (0.75-0.96) | 0.007 |
| Number of sex partners in prior 6 months |  |  |
| None | Reference |  |
| One | 1.23 (1.11-1.35) | <0.001 |
| Two or more | 1.40 (1.20-1.63) | <0.001 |
| Recent HIV-positive injection or sex partner | 1.40 (0.95-2.05) | 0.087 |
| Ever sex with a man (men only) | 1.01 (0.84-1.23) | 0.885 |
| Injection in prior 6 months |  |  |
| None | Reference |  |
| Less than daily | 0.71 (0.62-0.81) | <0.001 |
| Daily | 1.06 (0.92-1.22) | 0.388 |
| Shared needle/syringe in prior 6 months | 1.72 (1.55-1.92) | <0.001 |
| Hazardous alcohol use^2^ | 1.48 (1.34-1.63) | <0.001 |
| Unprotected sex in prior 6 months | 1.20 (1.10-1.32) | <0.001 |
| Sex work in prior 6 months | 1.33 (1.02-1.72) | 0.033 |
| Syringe service program use in prior 6 months | 1.27 (1.14-1.41) | <0.001 |
| Opioid agonist treatment in prior 6 months | 1.07 (0.96-1.20) | 0.200 |
| HIV test in prior 12 months | 1.04 (0.95-1.15) | 0.384 |
| Composite PWID stigma score (per 1-unit increase)^3^ | 1.03 (1.02-1.05) | <0.001 |
| Incarcerated in prior 6 months | 1.34 (1.14-1.58) | <0.001 |

CI: confidence interval; INR: Indian rupees

^1^ Multi-level logistic model with random intercept for site

^2^ Hazardous alcohol use defined as an Alcohol Use Disorders Identification Test (AUDIT) score ≥8

^3^ Injection drug use stigma calculated as the sum of four stigma sub-scales: experienced, vicarious, community and self-stigma with each sub-scale equally weighted. Stigma scores range from 0 to 20, with higher scores indicating higher levels of stigma.
