# Supplementary material for: Awareness of and willingness to use pre-exposure prophylaxis (PrEP) among people who inject drugs and men who have sex with men in India: Results from a multi-city cross-sectional survey
Source: PLoS One. 2021 Feb 25;16(2):e0247352. doi: 10.1371/journal.pone.0247352 (PMC7906475; doi:10.1371/journal.pone.0247352)
Supplement: S8 Table — (DOCX) [file pone.0247352.s010.docx]

**S8 Table**: Correlates of willingness to use oral pre-exposure prophylaxis among men who have sex with men (MSM) in India, **unweighted**

| **Correlate** | **Unadjusted Odds Ratio**  **(95% CI)^1^** | **P value** |
| --- | --- | --- |
| Age (per 5-year increase) | 0.99 (0.97-1.02) | 0.606 |
| Marital Status |  |  |
| Never married | Reference |  |
| Married/ living with partner/ long-term relationship | 0.95 (0.87-1.05) | 0.354 |
| Widowed/ divorced/ separated | 0.85 (0.66-1.10) | 0.223 |
| Sexual identity |  |  |
| Panthi^2^ | Reference |  |
| Kothi^2^ | 1.18 (1.04-1.34) | 0.012 |
| Double-Decker | 1.07 (0.95-1.21) | 0.238 |
| Gay/MSM | 1.57 (1.05-2.35) | 0.030 |
| Bisexual | 1.07 (0.88-1.30) | 0.518 |
| Education |  |  |
| Primary school or less | Reference |  |
| Secondary school or beyond | 1.01 (0.83-1.23) | 0.919 |
| Household income, tertiles (INR) |  |  |
| 0-11,000 | Reference |  |
| >11,000-20,000 | 1.02 (0.80-1.29) | 0.903 |
| > 20,000 | 0.93 (0.66-1.31) | 0.682 |
| Number of male partners in prior 6 months |  |  |
| One | Reference |  |
| Two to four | 0.98 (0.88-1.10) | 0.781 |
| Five or more | 1.33 (1.17-1.51) | <0.001 |
| Main male partner | 1.78 (1.55-2.04) | <0.001 |
| Type of anal sex with last 4 partners |  |  |
| No anal sex | Reference |  |
| Only penetrative | 0.76 (0.66-0.87) | <0.001 |
| Receptive (only or both penetrative and receptive) | 1.00 (0.88-1.15) | 0.977 |
| Unprotected anal intercourse in prior 6 months | 1.01 (0.92-1.11) | 0.789 |
| Sex work in prior 6 months | 1.07 (0.97-1.19) | 0.182 |
| Recent HIV-positive sex or injecting partner | 1.17 (0.78-1.73) | 0.448 |
| Symptoms of STI in prior 6 months | 1.68 (1.34-2.10) | <0.001 |
| Active syphilis infection | 1.12 (0.93-1.34) | 0.240 |
| HSV-2 positive | 1.05 (0.94-1.18) | 0.376 |
| Hazardous alcohol use^3^ | 1.13 (1.02-1.24) | 0.019 |
| Injected drugs in prior 6 months | 1.71 (0.89-3.27) | 0.107 |
| HIV test in prior 12 months | 1.25 (1.13-1.38) | <0.001 |
| Composite MSM stigma score (per 1-unit increase)^4^ | 1.04 (1.03-1.05) | <0.001 |

CI: confidence interval; INR: Indian rupees; STI: sexually transmitted infection

^1^ Multi-level logistic model with random intercept for site

^2^ *Panthi* and *kothi* refer to masculine and feminine sexual identities, respectively

^3^ Hazardous alcohol use defined as an Alcohol Use Disorders Identification Test (AUDIT) score ≥8

^4^ MSM stigma calculated as the sum of four stigma sub-scales: experienced, vicarious, community and self-stigma with each sub-scale equally weighted. Stigma scores range from 0 to 20, with higher scores indicating higher levels of stigma
